# Environmental toxicant glyphosate induces cardiotoxicity: New insights from network toxicology, integrated machine learning, molecular modeling and multidimensional bioinformatics analysis

**DOI:** 10.1097/MD.0000000000048974

**Published:** 2026-05-29

**Authors:** Mingze Sun, Yankai Wang, Renyang Tong, Ruiming Yan, Jun Pu

**Affiliations:** a State Key Laboratory for Systems Medicine for Cancer, Division of Cardiology, Shanghai Cancer Institute, Renji Hospital, School of Medicine, Shanghai Jiao Tong University, Shanghai, China; b Department of Respiratory and Critical Care Medicine, Beijing Institute of Respiratory Medicine and Beijing Chao-Yang Hospital, Capital Medical University, Beijing, China; c Department of Trauma Center, School of Medicine, Tongji Hospital, Tongji University, Shanghai, China.

**Keywords:** cardiotoxicity, glyphosate, hub genes, machine learning, molecular docking, network toxicology

## Abstract

Glyphosate is one of the world’s most widely used herbicide, yet the causal relationship between glyphosate and cardiotoxicity remains inadequate. To elucidate the cardiotoxic mechanisms of glyphosate, an integrative strategy was developed in this work, which combines computational toxicometry, predictive machine learning, and atomic-level molecular modeling. Initially, glyphosate’s systematic toxicological profile was evaluated using ProTox-3.0 and ADMETlab 3.0. Candidate genes associated with glyphosate were obtained from 3 separate databases. Target genes associated with cardiotoxicity were integrated from 2 databases. Intersection analysis identified 113 core candidate genes associated with glyphosate and cardiotoxicity. Functional enrichment-related analyses were employed and revealed that the target genes were significantly involved in key biological processes such as ERBB3 signaling pathways. In order to refine the candidate genes to a more precise gene pool, net topology analyses were employed and identified 16 key feature genes. We subsequently integrated machine learning algorithms and validated the results using the GEO database, ultimately identifying 4 hub genes, AKT1, IL1B, BRCA1, and PTGS2. Finally, we applied molecular docking analysis and revealed strong binding affinities, which suggested direct interaction mechanisms in glyphosate-induced cardiotoxicity. In summary, our study establishes a comprehensive mechanistic framework for glyphosate-induced cardiotoxicity.

## Graphical abstract

**Figure:**
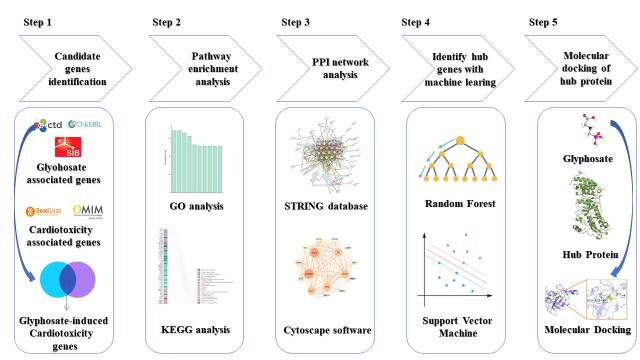

## 1. Introduction

Glyphosate (*N*-(phosphonomethyl)glycine) is the world’s most widely used broad-spectrum systemic herbicide. It maintains this dominant market position owing to its high efficacy against perennial weeds across agricultural and non-cropland settings.^[1]^ Due to its significant role in agriculture, the use of glyphosate is characterized by an exponential growth trajectory. This kind of abuse directly leads to its ubiquitous presence in soil, water systems, and the human food chain.^[2]^ Globally, approximately 850 thousand tons of chemical agents are released into the environment every year, particularly during soil preparation, preharvest desiccation, and storage. Despite achieving significant results in mitigating weed interference, the abuse of herbicides has also led to a series of environmental problems and cause extensive environmental contamination.^[3]^ In 2015, the World Health Organization’s International Agency for Research on Cancer classified it as “probably carcinogenic to humans” (Group 2A). However, accumulating evidences have illustrated that glyphosate can lead to acute and chronic impairment of vital organ functions, underscoring that its toxicological profile is not simply restrict in carcinogenicity alone.^[4]^ Currently, more and more studies have indicated that exposure to glyphosate-based herbicides can trigger a wide spectrum of biological disturbances, such as oxidative stress, endocrine disruption, and organ-specific impairment.^[5-7]^

Cardiotoxicity is defined as cardiac dysfunction resulting from either electrophysiological disturbances or direct myocardial tissue damage. This cardiac injury can induce systolic function and finally could destroys the heart’s capacity to sustain adequate systemic perfusion.^[8]^ Chronic or acute cardiotoxicity can trigger a cascade of adverse biological injuries, particularly in myocardial inflammation and cardiac rhythm disturbances, which can further destroy heart function.^[9,10]^ Prolonged chemical exposure can lead to chronic heart injuries, and this kind of injury is characterized by permanent structural damage to the myocardium. This persistent injury underscores the heart’s limited regenerative capacity in the face of sustained environmental insult.^[11,12]^ Currently, researchers have begun to explore the causal relationships between glyphosate exposure and specific cardiac pathologies. For example, the research on native amphibians and zebrafish embryos has reported that when exposed to glyphosate-based herbicides, the level of oxidative stress biomarkers increased significantly, and thus led to a significant reduction in heart rate.^[13]^ In another investigation, researchers reported that glyphosate exposure initiates a cascade of detrimental effects, spanning from morphological cardiac malformations to biochemical imbalances.^[14]^ However, Current toxicological studies still have limitations, which can be summarized into 3 points: first, conventional research often focuses on a single gene or single pathway; however, this kind of research often ignores the complex, system-wide BP which underlie disease progression. Second, current studies usually ignore the interrelationships between different pathways. The fact is that the pathways interact with each other, thereby mediating the occurrence of the disease, and this could not clarify the disease pathogenesis. Third, conventional experimental models often suffer from limited predictive power for multitarget effects and low throughput. This directly disturbs biomarker identification and decreases the prediction efficacy of timely therapeutic interventions.^[15,16]^ Taking all the limitations into consideration, current studies inevitably obscure how glyphosate induces cardiotoxicity. Network toxicology has emerged as a powerful, holistic approach, enabling the systematic identification of key nodes and signaling hubs involved in environmentally induced toxicities.^[17-20]^ Network toxicology studies have the following advantages: first, network toxicology can help the researchers adopt a broader perspective in disease pathogenesis. By constructing a comprehensive “pollutant-target-pathway” interactome, it provides a systemic visualization of glyphosate’s impact on the cardiac landscape, moving beyond the constraints of analyzing isolated molecular components.^[21]^ Second, this methodology could provide a robust framework for exploring complex pathway crosstalk and multi-target synergies. It can integrate diverse signaling cascades and merge as a whole network to clarify how glyphosate induces cardiotoxicity.^[22]^ Third, network toxicology focuses on the identification of critical hub genes and novel biomarkers. By integrating high-throughput computational modeling and virtual screening, network toxicology can predict the crucial hub targets for subsequent validation, thereby offering a highly efficient and cost-effective strategy to investigate glyphosate-induced cardiotoxicity.^[23,24]^

To elucidate the mechanism by which glyphosate mediates cardiotoxicity. We systematically mapped the toxicological interactome of glyphosate onto cardiotoxicity-specific signatures, and we integrated network toxicology, bioinformatic analysis, machine learning, and molecular docking. Collectively, our results establish a robust theoretical scaffold and intricate regulatory circuits of glyphosate, and establish a robust theoretical foundation for further experimental validation or assessment. These insights pave the way for environmental risk assessments and clinical strategies to prevent contaminant-induced cardiovascular disorders.

## 2. Materials and methods

### 2.1. Initial exploration of glyphosate toxicity

The structural information and the SMILES string of glyphosate were downloaded from the PubChem database. To evaluate its toxicological profile, glyphosate’s structural messages were subsequently submitted to 2 predictive databases: ProTox-3.0 and ADMETlab 3.0. Both tools could provide a relatively multifaceted assessment of glyphosate’s toxicity, offering a systematic overview of its potential biological risks.

### 2.2. Collection of glyphosate target

To obtain the potential targets of glyphosate, we utilized 3 databases: the Comparative Toxicogenomics Database, ChEMBL database and the Swiss Target Prediction database. Then, we restricted the species to “Homo sapiens” to ensure the targets we obtained were biologically interpretable. If there were some targets do not match the names of genes, the UniProt database was utilized to verify and map the identified targets, ensuring all entries we obtained were aligned with their recognized official gene symbols. Finally, we integrated all genes obtained from the databases and finally got a gene set related to potential targets of glyphosate.

### 2.3. Identification of potential cardiotoxicity-related targets

To identify potential cardiotoxicity-related targets, we set “Cardiotoxicity” as the primary keyword and conducted a comprehensive search across the OMIM and GeneCards databases. To ensure the genes we chose were highly associated with cardiotoxicity, we applied a stringent filtering criterion: we calculated the Relevance Score, then we set the threshold at the median value. We only chose the genes with scores equal to or exceeding the relevance score before for further analysis. Subsequently, the target genes obtained from 2 databases were merged. The redundant genes were removed, and the reliable gene set was defined as being related to cardiotoxicity.

Furthermore, intersection analysis with a Venn diagram was applied to visually represent the targets that linked to both glyphosate and cardiotoxicity. The target genes that underwent the analyses above were defined as the initial target genes which responsible for glyphosate-induced cardiotoxicity. These targets were given priority in the following system-level investigation.

### 2.4. GO and KEGG pathway enrichment analysis of the intersection target

To investigate the functions of the target genes we identified above, we performed a comprehensive enrichment analysis. This approach elucidated the BP and functional ontologies associated with the overlapping gene set.^[25]^ Initially, target genes underwent functional annotation via Gene Ontology (GO), in this analysis, intersection genes were systematically classified across 3 core dimensions: physiological pathways (Biological Process), subcellular localization (Cellular Component), and biochemical activities (Molecular Function). Furthermore, KEGG-based enrichment analysis facilitated the identification of essential biological circuits through which chemical insults integrate with host leukocyte responses to drive cardiotoxic outcomes.

The GO enrichment pipeline was implemented in R (version 4.4.1; The R Foundation for Statistical Computing) with 2 core R packages: the “clusterProfiler” and ‘org.Hs.eg.db’ packages. Statistical rigor was maintained by enforcing a significance threshold where both *P*- and *q*-values were restricted to <.05. Following functional profiling, the top ten most significant terms within each GO category were identified.^[26]^ These top pathways were prioritized for subsequent visualization and mechanistic interpretation. For the identification of therapeutic pathways, KEGG enrichment was executed using the “clusterProfiler” R package, with “org.Hs.eg.db’ facilitating the gene symbol-to-Entrez ID conversion. Statistical filtration was strictly governed by a *P*-value cutoff of .05 and a *q*-value cutoff of 0.05, ultimately prioritizing the top 20 signaling routes to highlight the primary toxicological mechanisms.

### 2.5. Protein–protein interaction network construction and hub gene selection

A protein*–*protein interaction (PPI) architecture was established to characterize the global landscape of target associations, facilitating the prioritization of core hub genes for subsequent mechanistic deconvolution and molecular modeling.^[27]^ To decipher the functional interplay among the identified targets, a global PPI network was initially visualized using the STRING database (version 12.0; Structural and Computational Biology Group, EMBL), to enhance the robustness of the derived network and mitigate potential noise, a high-stringency confidence score of 0.700 was enforced as the minimum interaction threshold. Subsequently, to make the results of the PPI network analysis more reliable, we downloaded the results from STRING (TSV format), then put the file into Cytoscape (version 3.9.1; The Cytoscape Consortium) for further and deeper analysis. To pinpoint the most important hubs throughout the whole protein interaction network, when we applied the analysis in Cytoscape software, a multiparameter screening strategy was set. This strategy strictly filtered the candidate targets with 3 centrality measures: degree, closeness, and radiality. Only nodes scoring above the median for each metric were retained for the following analysis.

### 2.6. Machine learning-based core gene selection

To further validate the hub genes identified in PPI network analysis, we searched an independent external transcriptomic GEO dataset (GSE261326). In this dataset, we extracted the samples which belonged to “Untreated” and “Dox,” representing the control groups and cardiotoxicity groups. Additionally, we also extracted the expression profiles of the identified hub genes, and a new validation matrix was established. And we implemented 2 distinct machine learning frameworks: Random Forest (RF) and Support Vector Machine (SVM).

RF provided a robust ranking of genes based on the Mean Decrease Gini metric. This approach effectively identifies the most contributory features to classification accuracy, even as it filters out noise inherent in high-dimensional data.^[17,28]^ Through SVM implementation, we derived the optimal separating hyperplane that maximized the margin between groups. Genes associated with the highest weight coefficients were thereby identified as key contributors to precise group discrimination.^[29,30]^ In RF, the ensemble was built with a fixed random seed (we set the random seed to 123). We ranked the 16 candidate genes by their importance for node purity in classification, using the Mean Decrease Gini metric to quantify each gene’s contribution. To extract the weight coefficients, a linear kernel was applied in the SVM analysis. The absolute values of these weights were used to evaluate the influence of each gene on the construction of the optimal separating hyperplane.

To ensure the robustness of our selection, we performed an intersection analysis of the top 5 genes ranked by the RF and SVM algorithms. The overlapping genes were subsequently defined as the core target signatures for further analysis.

### 2.7. Molecular docking analysis of the core target genes

To investigate the binding affinity and interaction modes between glyphosate and the identified core targets associated with cardiotoxicity (AKT1, Mpro, SIRT2, and IL1B), molecular docking simulations were performed using the AutoDock Vina (version 1.1.2; The Center for Computational Structural Biology, Scripps Research Institute, La Jolla) software. The chemical structure of glyphosate was retrieved from the PubChem database and subsequently converted into 3D format. The crystal structures of the receptors were retrieved from the RCSB Protein Data Bank (PDB IDs: 4GV1, 5R8G, 4IGK, and 5R85). Additionally, the species of receptors was restricted to Homo sapiens to ensure biological relevance and interpretability of the toxicological results. Furthermore, only crystal structures with a resolution of <2 Å were selected to provide high-fidelity atomic coordinates for docking accuracy. During protein preprocessing in PyMOL, water molecules, ions, and co-crystallized original ligands were removed, followed by the addition of polar hydrogens and charges.

To get the exact localization of the binding pockets for each target gene (AKT1, BRCA1, IL1B, ERBB2), we set the docking grid box centered on the ligand of the respective co-crystallized inhibitors to ensure biologically relevant binding poses. We set the size of the grid box to 20 Å ×20 Å ×20 Å, and the exhaustiveness parameter was set to 12 to balance computational efficiency and search thoroughness.

Molecular docking simulations were conducted using the Lamarckian Genetic Algorithm. To make sure the results have high convergence and reproducibility, each simulation comprised 50 independent runs. The resulting poses were then evaluated and ranked by their calculated binding energy (kcal/mol). More negative values mean a stronger predicted binding affinity. To visualize the results of the molecular docking, we used PyMOL software. In the meantime, we also characterized the non-covalent interactions, including hydrogen bonds, hydrophobic contacts, and van der Waals forces.

### 2.8. Ethical statement

The data utilized in this study were obtained from public databases. Therefore, ethical approval and informed consent were not required as all data are de-identified and publicly accessible.

## 3. Results

### 3.1. Toxicological analysis of glyphosate

**Figure 1.**
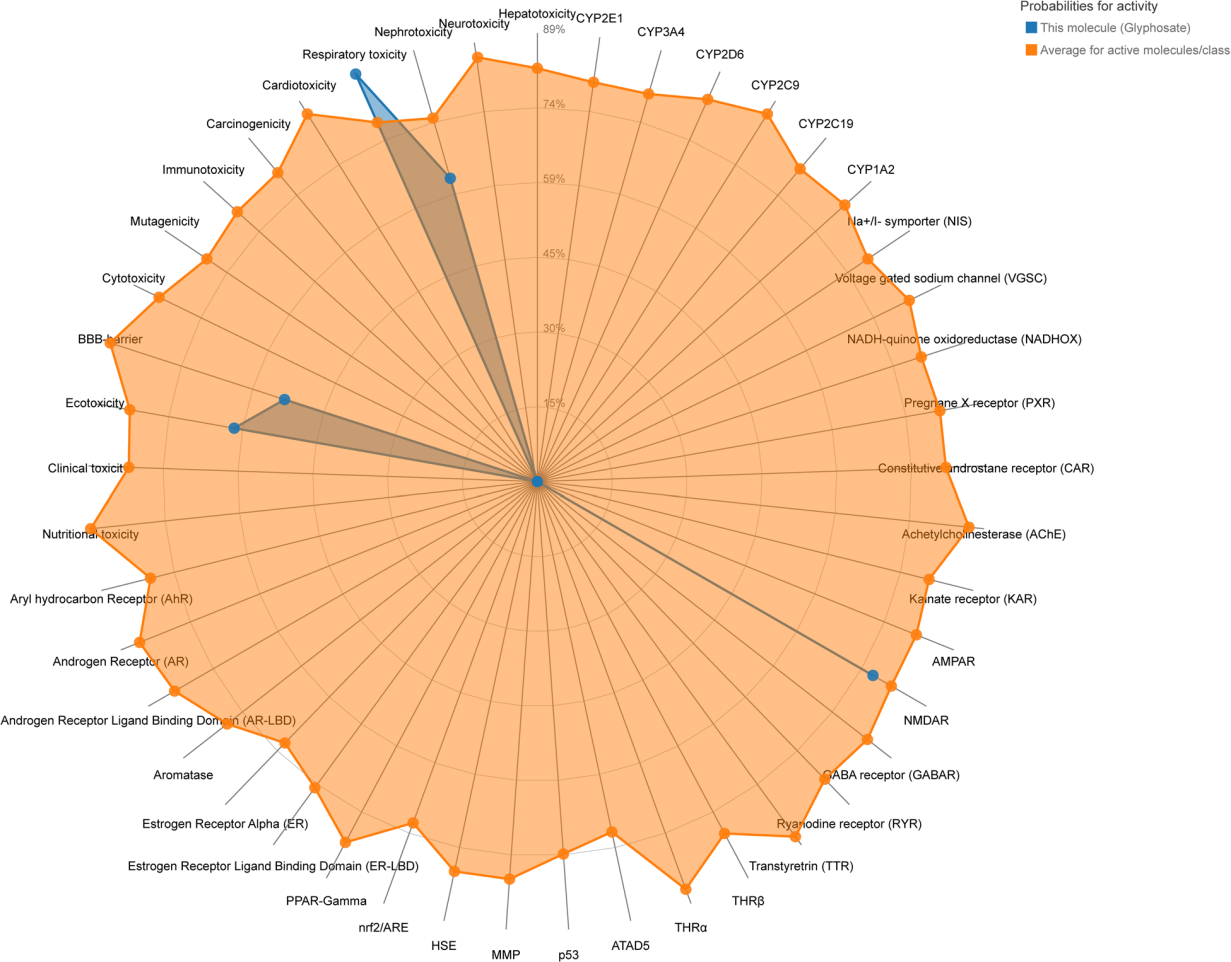
Radar chart of the toxicity of glyphosate.

The structural attributes and SMILES string of glyphosate were sourced from the PubChem database, with the detailed chemical profiles summarized in Table 1. Through integrating the toxicological profiles of glyphosate by ProTox-3.0 and ADMETlab 3.0, we obtained an initial summary of glyphosate toxicity (Fig. 1, Tables S1 and S2, Supplemental Digital Content). The computational analysis revealed that glyphosate possesses significant potential for localized and systemic toxicity. According to ADMETlab 3.0 and ProTox-3.0, glyphosate exhibited high probabilities for eye toxicity and respiratory toxicity, alongside a strong likelihood of drug-induced nephrotoxicity. According to the result of ADMETlab 3.0, glyphosate also exhibited a demonstrable correlation with cardiotoxicity.

**Table 1:**
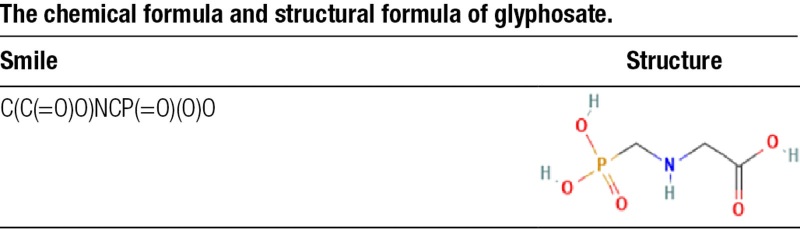
The chemical formula and structural formula of glyphosate.

### 3.2. Identification of the targets of glyphosate-induced cardiotoxicity

**Figure 2.**
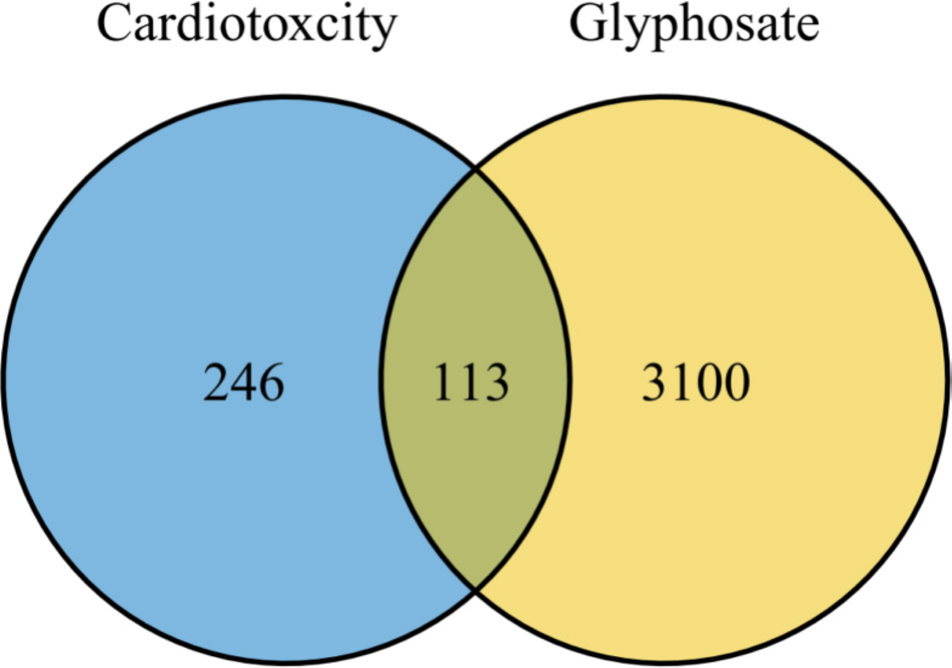
Venn diagram of glyphosate and cardiotoxicity target genes.

In our study, we identified 3213 target genes associated with glyphosate from Comparative Toxicogenomics Database, ChEMBL database and Swiss Target Prediction database. At the same time, 359 target genes of cardiotoxicity were chosen from the OMIM and GeneCards databases. To identify high-confidence genes involved in glyphosate-mediated cardiotoxicity, an intersection analysis of the 2 gene sets was performed using a Venn diagram. Consequently, a total of 113 overlapping genes were identified and defined as the core candidates closely associated with cardiotoxicity (Fig. 2).

### 3.3. Functional enrichment analysis of glyphosate-induced cardiotoxicity target genes

**Figure 3.**
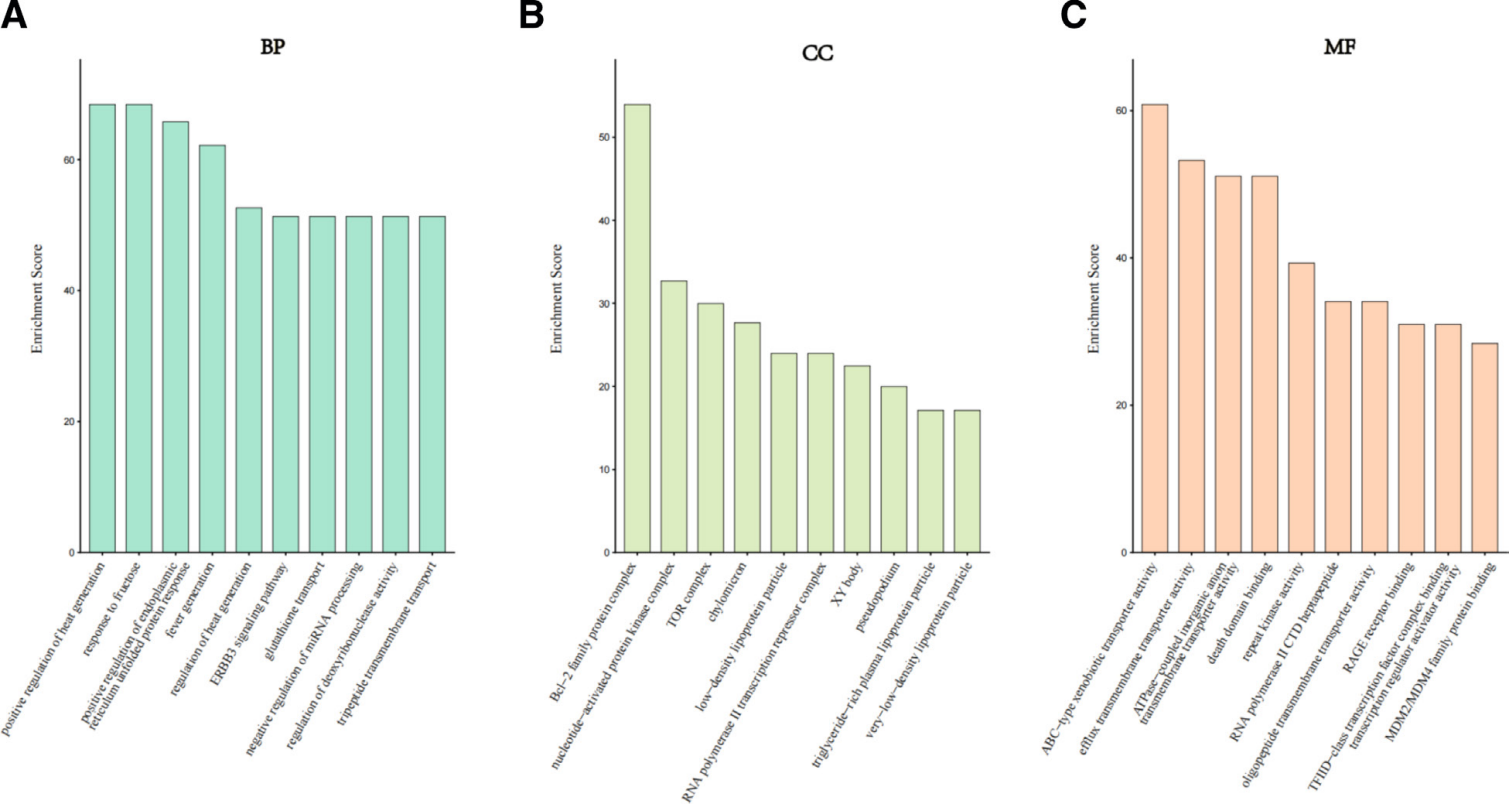
GO enrichment analysis of intersection target genes. GO enrichment analysis of intersection target genes. (A) Biological processes enrichment analysis of 113 intersection genes; (B) Cellular components enrichment analysis of 113 intersection genes; (C) Molecular functions enrichment analysis of 113 intersection genes. GO = Gene Ontology.

To further elucidate the biological functions and potential mechanisms of the 113 core target genes, GO functional enrichment analysis was performed, encompassing biological processes (BP), cellular components (CC), and molecular functions (MF). In the BP compartment, genes primarily participate in the endoplasmic reticulum unfolded protein response and heat generation, suggesting that glyphosate triggers robust cellular stress and energy metabolism disturbances. In the CC part, Candidate genes are predominantly localized in the Bcl-2 family protein complex and the TOR complex, highlighting their roles in regulating cardiomyocyte apoptosis and growth signaling. As for MF, genes were significantly enriched in ABC-type xenobiotic transporter activity and death domain binding, which may illustrate that after exposure to glyphosate, cellular apoptosis may be activated (Fig. 3).

**Figure 4.**
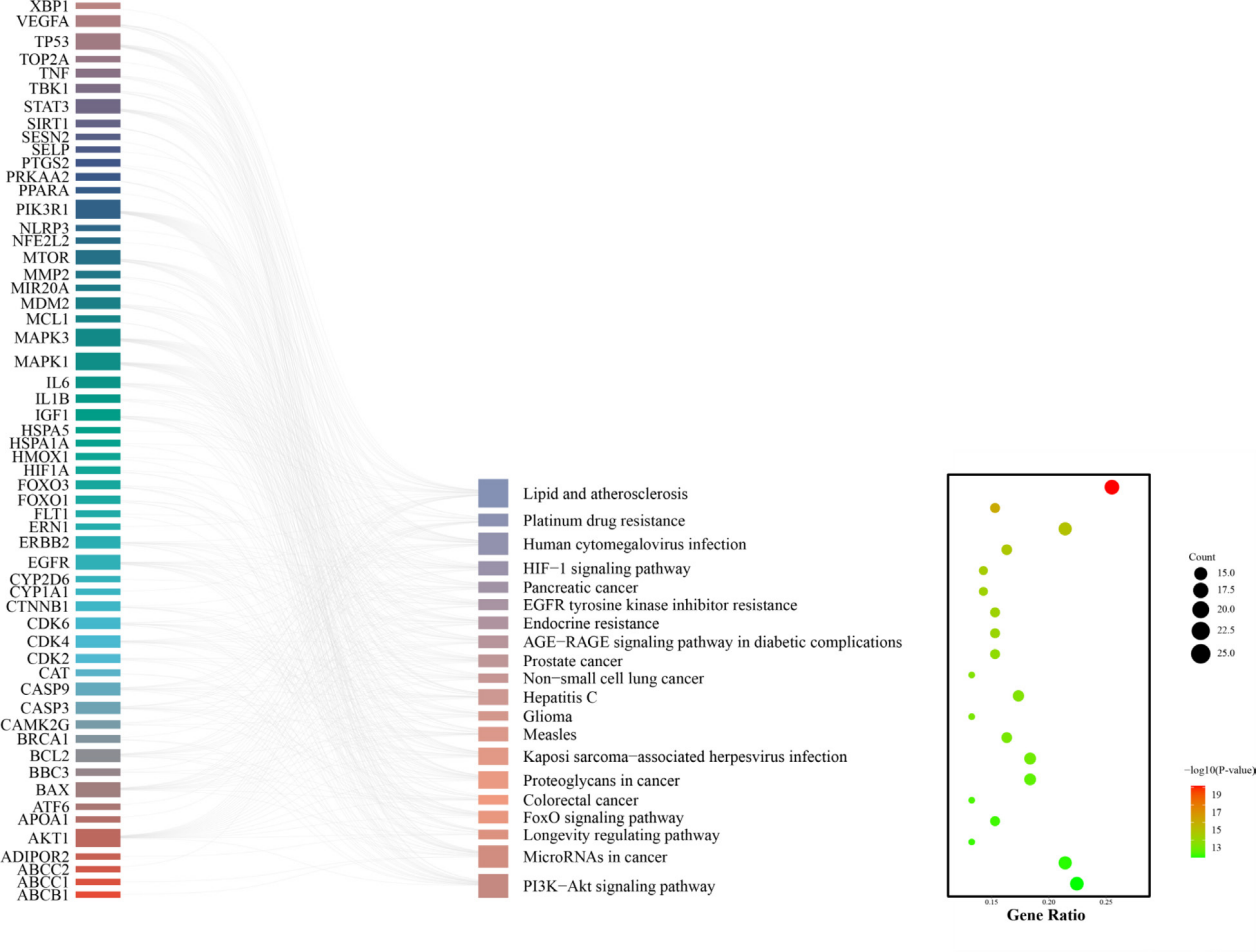
Enrichment of KEGG pathway for intersection genes Sankey and bubble diagrams. KEGG = Kyoto Encyclopedia of Genes and Genomes.

In order to investigate the systemic impact of glyphosate, we also conducted KEGG pathway enrichment analysis. In our results, we revealed the top 20 signaling pathways significantly associated with core target genes, as illustrated in the enrichment network. These pathways are primarily involved in cell survival, environmental stress response, and chronic cardiovascular pathologies (Fig. 4).

### 3.4. The potential target interaction network and the acquisition of core genes

**Figure 5.**
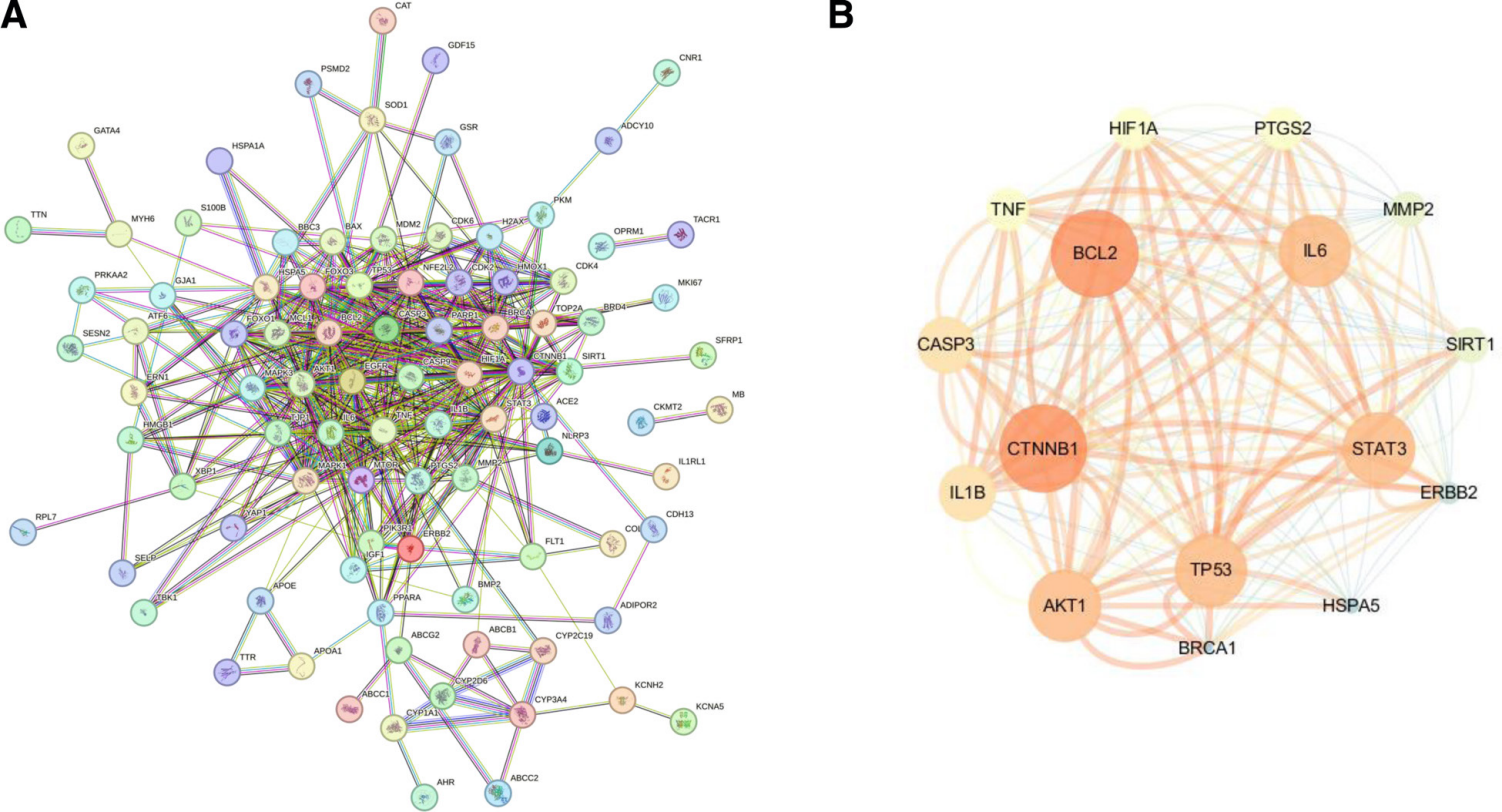
Enrichment of KEGG pathway for intersection genes Sankey and bubble diagrams. Protein–protein interaction (PPI) network analysis of intersection genes. (A) The PPI network of 113 intersection genes using the STRING database, highlighting the functional connectivity of glyphosate-induced- cardiotoxicity genes; (B) Topological analysis with Cytoscape software, hub genes were isolated after filtering based on betweenness, closeness, and degree. KEGG = Kyoto Encyclopedia of Genes and Genomes, PPI = Protein–protein interaction.

To further investigate the functional synergism among the 113 candidate targets, a protein–protein interaction (PPI) network was constructed using the STRING database and visualized via Cytoscape. The initial network results produced by STRING database illustrated a highly interconnected architecture, which contained multiple nodes and complex functional clusters. This indicated that glyphosate-mediated myocardial toxicity is a complex regulatory mechanism involving multiple molecular networks. To further isolate the critical biological targets related to glyphosate-induced cardiotoxicity within this complex network, we downloaded the result in STRING analysis and put them into the Cytoscape software for subsequent analysis. This analysis identified the 16 most critical hub genes, which constitute the functional epicenter of the glyphosate-induced cardiotoxicity network. These core genes include BCL2, CTNNB1, TP53, AKT1, IL6, STAT3, CASP3, TNF, HIF1A, PTGS2, MMP2, SIRT1, ERBB2, IL1B, BRCA1, and HSPA5. As the results illustrated in the hub gene subnetwork in Figure 5B, there was a close interaction between apoptotic regulators (BCL2, TP53) and pro-inflammatory cytokines (IL6, TNF), which indicated that activation of cell death and inflammatory pathways may be involved in glyphosate-induced cardiotoxicity. These 16 hub genes were isolated and were ready for subsequent analysis and validation, as they likely play an important role in the pathophysiological mechanism through which glyphosate mediates its cardiotoxic effects (Fig. 5A, B).

### 3.5. Machine learning

To further refine the 16 candidate targets and identify the most robust biomarkers for glyphosate-induced cardiotoxicity, we downloaded an external transcriptomic dataset (GSE261326) for further analysis and validation. In this GEO dataset, we extracted 2 groups, “Untreated” and “Dox,” which represented control and cardiotoxicity, separately. Then we implemented 2 distinct machine learning algorithms, RF and SVM, which were used for feature importance ranking.

**Figure 6.**
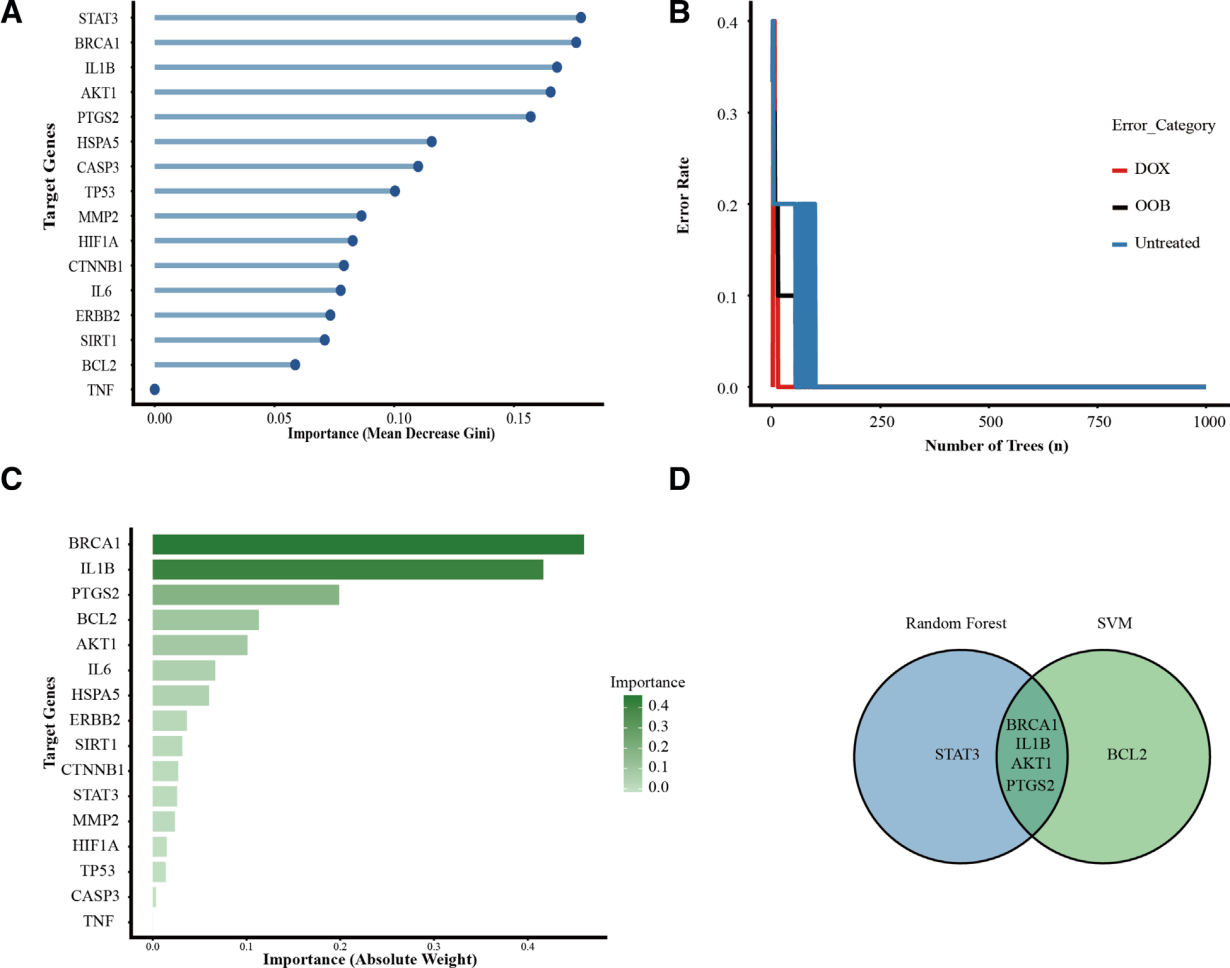
Identification of hub genes via integrated machine learning. Candidate gene selection using RF and SVM algorithms. (A) Ranking of target genes based on their importance scores (Mean Decrease Gini) calculated by the RF; (B) the error rate curve of the RF classifier. The black line represents the out-of-bag (OOB) error, while the red and blue lines denote the error rates for the DOX-induced cardiotoxicity group and the untreated control group, respectively; (C) distribution of gene importance weights determined by the SVM algorithm. Genes are ranked by their absolute weight values; (D) a Venn diagram illustrating the intersection of candidate genes identified by both RF and SVM models. Four hub genes, including BRCA1, IL1B, AKT1, and PTGS2, were consistently identified by both machine learning frameworks and selected for further downstream analysis. DOX = Doxorubicin, OOB = out-of-bag, RF = Random forest, SVM = support vector machine,

We extracted the transcriptomic profiles of 16 hub genes identified in PPI network analysis, a smaller but more specific dataset was established for subsequent machine learning analysis. The RF model was trained, and the feature importance was quantified via the Mean Decrease Gini index. The result of the importance plot is shown in Figure 6. STAT3, BRCA1, IL1B, AKT1, and PTGS2 were the top 5 most significant variables contributing to the classification of cardiotoxicity versus the control group. These 5 genes yielded importance scores above 0.15, underscoring their potential role in the pathogenesis of cardiotoxicity (Fig. 6A, B). Simultaneously, we applied a linear kernel SVM model to evaluate the absolute weight coefficients of the target genes. SVM was more inclined to rank the importance of genes, 1 that complemented other metrics and thus made the results more convincing. According to the results produced by the SVM weight analysis. BRCA1, IL1B, PTGS2, BCL2, and AKT1 were identified as the top 5 key features, which had the highest absolute weights. Among these 5 genes, BRCA1 and IL1B showed absolute weights, both of which exceeded 0.4, which illustrated their importance in the definition of the optimal separating hyperplane.

After conducting 2 distinct machine learning analyses, we intersected the top 5 genes from both models. The results showed that 4 consensus biomarkers were finally identified: BRCA1, IL1B, PTGS2, and AKT1. These 4 genes, identified as high-confidence diagnostic signatures, were considered as the key targets for subsequent molecular docking to explore their direct interactions with glyphosate.

### 3.6. Molecular docking

**Figure 7.**
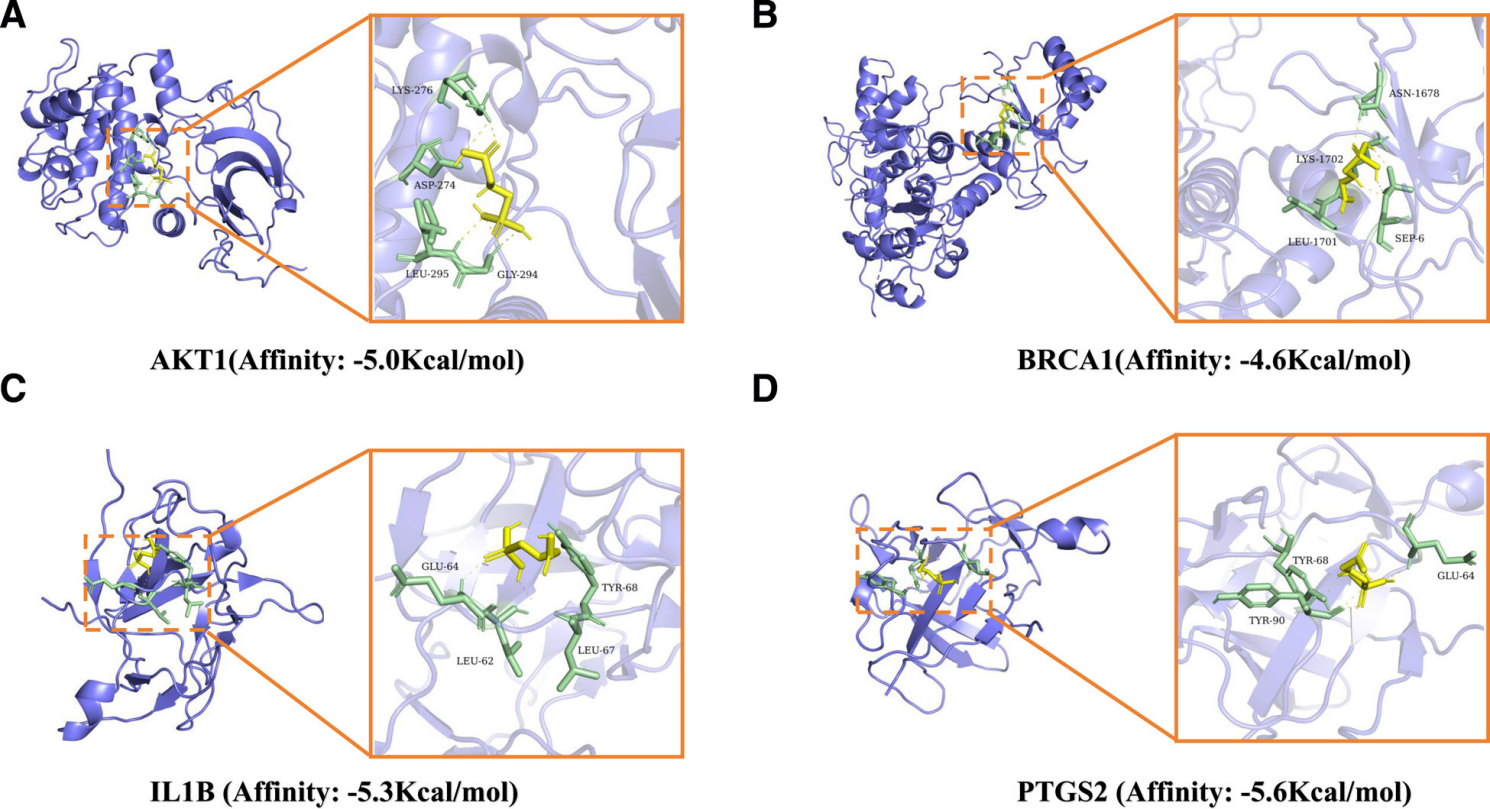
Molecular docking simulations of glyphosate with hub proteins. Molecular docking simulations of glyphosate with hub proteins. (A) Glyphosate binding to AKT1 with a binding affinity of −5.0 kcal/mol; (B) glyphosate binding to BRCA1 with a binding affinity of −4.6 kcal/mol; (C) glyphosate binding to IL1B with a binding affinity of −5.3 kcal/mol; (D) glyphosate binding to PTGS2 with a binding affinity of −5.6 kcal/mol.

Molecular docking simulations were performed to evaluate the binding characteristics between glyphosate and the 4 identified hub proteins (AKT1, BRCA1, IL1B, and PTGS2). Molecular docking analysis was performed with AutoDock Vina (v1.1.2). Results of energetically favorable binding affinities were shown in Figure 7, the binding energies ranged from −4.6 to −5.6 kcal/mol (Fig. 7). As shown in the results, glyphosate fits precisely in the pockets of these proteins and forms critical hydrogen bonds and hydrophobic interactions with key amino acid residues. Take PTGS2 as an example, glyphosate have the interaction with TYR-90, TYR-68, and GLU-64. These negative binding energy values illustrated that glyphosate can associate with each core protein we identified, suggesting that these targets are critical to the molecular network governing glyphosate-induced cardiotoxicity.

## 4. Discussion

Nowadays, glyphosate is one of the most extensively applied in agriculture, primarily due to the widespread adoption of glyphosate-resistant crops. Although its main mechanism involves in the disruption of the shikimate pathway in plants, a growing body of research illustrated that glyphosate may play an important role in nontarget toxicity in mammalian systems, with particular attention to its possible association with cardiovascular dysfunction.^[31,32]^ For instance, in 1 study, researchers utilized human cardiomyocytes and exposed them to glyphosate; the results showed that severe cardiac impairments, such as the blockade of cardiac calcium channels, are primarily attributable to exposure to glyphosate.^[31]^ In another study, by utilizing the vertebrate developmental models, the researchers reported that glyphosate could significantly perturb the development of myocardial precursors and induce structural abnormalities in the heart.^[32]^ However, current studies focused on the causal relationship between glyphosate and cardiotoxicity still exist some limitations: first, although the current research has elucidated the association between glyphosate and myocardial toxicity at certain targets and pathways, the precise molecular targets and systemic regulatory networks through which glyphosate interacts with cardiac tissues remain inadequately defined. Second, current research has largely focused on macro-level physiological phenomena or early developmental toxicity, leaving the precise molecular targets and the systemic regulatory networks of glyphosate in the adult heart inadequately characterized.

To bridge these gaps, in our study, we implemented an integrated framework encompassing network toxicology, machine learning, and molecular modeling to systematically explore the cardiotoxic mechanisms of glyphosate. Specifically, we first performed a comprehensive cross-platform database screening to identify candidate genes associated with both glyphosate exposure and myocardial toxicity. Through this step, we can obtain the most comprehensive molecular targets that are related to both glyphosate and cardiotoxicity. Furthermore, in order to have a comprehensive insight into the functional landscapes of the genes we initially chose, bioinformatic analyses such as GO and KEGG enrichment analyses were conducted, and the results revealed that candidate genes are significantly involved in pathways such as the regulation of heat generation and fever generation pathway. Subsequently, PPI analysis was employed to elucidate the complex functional crosstalk among differential genes and to identify key hub proteins that drive the molecular response. To validate and enhance the precision of our results produced in PPI analysis, machine learning algorithms, which included LASSO and SVM, were employed to cross-validate these hub genes within the GEO transcriptomic dataset. Finally, we utilized molecular docking simulations to provide atomic-level evidence of the binding affinity between glyphosate and its key targets, such as AKT1, IL1B, BRCA1 and PTGS2. Taking all the results above into consideration, this study offers a more definitive and multidimensional assessment of glyphosate’s intrinsic cardiotoxicity risks.

We initially screened the potential targets of glyphosate in our study, and we subsequently leveraged 2 databases, ProTox-3.0 and ADMETlab 3.0. These 2 databases could give us a comprehensive groundwork for understanding the multi-organ risks associated with glyphosate exposure. Although glyphosate is widely perceived as a low-toxicity herbicide,^[33]^ when we integrated the results from 2 databases, the results of computational analyses illustrated that glyphosate may induce systemic injury, such as high probabilities for respiratory, eye, and renal toxicities. According to the results produced by ADMETlab 3.0, the relationship between cardiotoxicity and glyphosate exposure has been proven (Table S2, Supplemental Digital Content). Which highlighted the necessity to further investigate the underlying cardiovascular mechanisms. A Venn diagram-based intersection analysis was employed to move beyond general toxicity and identify the precise molecular drivers. A Venn diagram-based intersection analysis was employed to move beyond general toxicity and identify the precise molecular drivers. By cross-referencing 3213 glyphosate-associated genes with 359 cardiotoxicity-specific targets, we successfully isolated 113 core candidate genes. Unlike previous research that might focus on broad environmental impacts, our study more precisely explored the “glyphosate-cardiovascular” axis, and these 113 intersection genes play an important role in building the crucial bridge for the subsequent bioinformatic analyses, providing a comprehensive landscape for isolating hub genes. These genes are also key determinants of the pathological outcomes in glyphosate-induced cardiotoxicity.

Glyphosate-induced cardiotoxicity is a complex and a multifaceted process rather than single-gene alterations or simple pathway changes. To get a comprehensive understanding of the occurrence and development of a disease, bioinformatic analysis such as GO enrichment analysis could overcome this limitation. Our GO enrichment data provides a systemic overview of this complexity, which illustrates that target genes are significantly involved in the positive regulation of heat generation and response to fructose. The dysregulation of thermogenic pathways often reflects mitochondrial uncoupling; as a result, this will lead to an increased level in reactive oxygen species within cardiomyocytes.^[34]^ If coupled with the dysfunction in fructose metabolism, this will further exacerbate oxidative stress and trigger apoptotic cascades that underpin glyphosate-induced heart injury. ERBB3 signaling pathway is also enriched in the analysis, which suggests that glyphosate may disrupt the myocardial redox balance and growth factor signaling.^[35]^ In the CC analysis, the Bcl-2 family protein pathway is significantly enriched. The Bcl-2 family protein plays an important role in myocardium apoptotic balance.^[36]^ For example, it can trigger the permeabilization of the mitochondrial outer membrane, which can lead to the release of cytochrome c and finally drive the apoptosis of the cardiomyocytes.^[37]^ In terms of MF, there is a high enrichment score in ABC-type xenobiotic transporter activity. This implies that glyphosate may destroy the heart’s primary cellular defenses and will overwhelm ABC transporter function.

KEGG pathway analysis delineates the systemic cardiovascular impact of glyphosate, revealing robust functional clustering in atherosclerosis-related lipid metabolism and the PI3K-Akt pathway. This implicates glyphosate in promoting plaque formation and vascular inflammation – core processes in chronic cardiotoxicity. We also find that HIF-1 and FoxO pathways are also enriched, which indicates that glyphosate-induced-cardiotoxicity may be related to pseudo-hypoxia and oxidative stress. Crucially, the convergence of these pathways on central hub genes (e.g., AKT1, IL1B) demonstrates that glyphosate acts not on a single target, but via an integrated network that dysregulates metabolic sensing, inflammatory responses, and survival signaling. This network-level disruption ultimately drives pathological outcomes such as arrhythmias and contractile dysfunction.

PPI network analyses reveal that glyphosate-induced cardiotoxicity is a complex network, rather than a simple gene alteration or single pathway (Fig. 5). By applying topological analysis, we identified a higher-confidence subnetwork consisting of 16 core hub genes. To validate the selection of hub genes and enhance the diagnostic precision of our toxicological model, we integrated 2 distinct machine learning algorithms: RF and SVM. While PPI network analysis identifies central nodes based on physical interactions, machine learning evaluates these genes’ predictive power within actual transcriptomic landscapes, such as the GEO dataset. We successfully isolated a high-fidelity gene signature consisting of AKT1, IL1B, BRCA1, and PTGS2 (Fig. 6D). Among these 4 genes, PTGS2 and IL1B act as a key role in inducing “cytokine storm-like” microenvironment within the heart. PTGS2 is a well-known catalyst for pro-inflammatory prostaglandins, which can exacerbate myocardial oxidative stress.^[38]^ When acting in concert with IL1B, a potent pyrogen and inflammatory orchestrator, these factors may trigger chronic low-grade inflammation, leading to the progressive fibrotic remodeling and rhythm disturbances often observed in herbicide-related cardiac pathologies.^[39]^ AKT1 is a master regulator of the PI3K/Akt pathway.^[40]^ AKT1 serves as the heart’s primary endogenous defense against apoptotic triggers.^[41]^ A downregulation or functional impairment of AKT1 effectively strips cardiomyocytes of their survival “safety net.” This vulnerability is particularly critical in the heart, where the limited regenerative capacity means that the loss of AKT1-mediated signaling can directly translate into a permanent reduction in contractile mass and the onset of heart failure.^[42]^ Traditionally studied in oncology,^[43]^ BRCA1’s role in the myocardium is increasingly recognized as a cornerstone of DNA damage repair.^[44]^ The involvement of BRCA1 suggests that glyphosate may exert genotoxic pressure on heart cells. If BRCA1-mediated repair mechanisms are overwhelmed or suppressed, the accumulation of double-strand breaks can stall the cell cycle or force cardiomyocytes into premature senescence, further compromising the structural integrity of the heart over time.^[45]^

The final part of our investigation, molecular docking simulations, provides the structural evidence for the interactions between glyphosate and the final-identified 4 key genes. Among these 4 candidates, PTGS2 shows the most robust binding affinity, with a binding free energy of −5.6Kcal/mol (Fig. 7D and Table 2). Detailed mapping of the PTGS2-glyphosate complex reveals that the herbicide anchors firmly within the catalytic pocket, primarily stabilized by critical hydrogen bonds with residues TYR-68 and GLU-64. This high thermodynamic stability suggests that glyphosate may act as a competitive ligand for PTGS2, potentially amplifying the inflammatory signaling pathways that drive chronic myocardial remodeling.^[46]^ Similarly, the binding profiles for IL1B (−5.3 Kcal/mol) and AKT1 (−5.0 Kcal/mol) underscore the multi-targeted nature of glyphosate’s toxicity (Fig. 7A, C and Table 2). In the case of AKT1, the molecular orientation of glyphosate is maintained through specific interactions with LYS-276 and ASP-274, sites that are often pivotal for the kinase’s phosphorylation and subsequent activation.^[47]^ The disruption of such a central survival node, coupled with the observed affinity for the DNA-repair protein BRCA1 (−4.6 Kcal/mol), points toward a cumulative failure of cellular defense mechanisms (Fig. 7B and Table 2).^[48]^

**Table 2:** Binding affinities of glyphosate with 4 core target proteins.

Target gene	PDB ID	Minimum binding energy
AKT1	4GV1	−5.0 kcal/mol
BRCA1	4IGK	−4.6 kcal/mol
IL1B	5R85	−5.3 kcal/mol
PTGS2	5R8G	−5.6 kcal/mol

PDB = Protein Data Bank.

In summary, the primary highlight of this study lies in its integrative multidimensional approach, which bridges the gap between massive bioinformatics screening and precise molecular docking validation. Unlike conventional reductionist studies that focus on single targets, we have successfully delineated a system-wide regulatory network involving AKT1, IL1B, BRCA1, and PTGS2, providing a more holistic understanding of how glyphosate undermines cardiac integrity. Furthermore, incorporating machine learning algorithms (RF and SVM) to refine our gene selection within external GEO cohorts significantly enhances the diagnostic reliability and translational potential of our findings.

However, several limitations should be acknowledged. Firstly, although our molecular docking simulations provide compelling structural evidence for glyphosate-protein interactions, these results remain computational predictions that require further validation through in vitro binding assays or site-directed mutagenesis. Secondly, while the identified hub genes and enriched pathways (such as Bcl-2 mediated apoptosis and ABC-type transport) offer plausible mechanistic insights, the precise causal sequence of these events in a living organism remains to be elucidated via animal models or primary cardiomyocyte experiments. Future research should focus on longitudinal studies to observe the chronic progression of these molecular shifts into clinical cardiac phenotypes.

## 5. Conclusion

Considering all the results, our study provides a comprehensive and multidimensional investigation of the mechanisms by which glyphosate induces cardiotoxicity. By integrating multi-database network toxicology, bioinformatic analyses, machine learning, and molecular docking analysis, our study has overcome the narrow scope of earlier research, which focused on a single target or simple pathway. Our results indicate that glyphosate specifically targets the heart. Rather than inducing generic stress, it mounts a structured attack on critical survival and inflammatory hubs, particularly disrupting the AKT1/IL1B axis. Specifically, results of molecular docking illustrate that glyphosate acts as a potential competitive inhibitor of 4 genes that are related to glyphosate-induced cardiotoxicity. These solid conclusions pave the way for further in vivo and in vitro experiment validations. In summary, this research establishes a clear molecular linkage between glyphosate exposure and cardiotoxicity. It provides a theoretical evidence base for refining environmental risk guidelines and cardioprotective strategies.

## Acknowledgments

The authors would like to express their sincere gratitude to all the colleagues and peers who provided valuable suggestions and technical assistance during this study.

## Author contributions

**Conceptualization:** Mingze Sun, Yankai Wang, Renyang Tong, Jun Pu.

**Data curation:** Mingze Sun.

**Funding acquisition:** Jun Pu.

**Investigation:** Mingze Sun.

**Methodology:** Ruiming Yan.

**Project administration:** Jun Pu.

**Supervision:** Yankai Wang, Renyang Tong.

**Validation:** Mingze Sun.

**Visualization:** Mingze Sun, Ruiming Yan.

**Writing – original draft:** Mingze Sun, Yankai Wang.

**Writing – review & editing:** Mingze Sun, Yankai Wang.




