# Supplementary material for: Environmental toxicant glyphosate induces cardiotoxicity: New insights from network toxicology, integrated machine learning, molecular modeling and multidimensional bioinformatics analysis
Source: Medicine (Baltimore). 2026 May 29;105(22):e48974. doi: 10.1097/MD.0000000000048974 (PMC13225543; doi:10.1097/MD.0000000000048974)
Supplement: Supplementary file 1 [file medi-105-e48974-s001.docx]

**Supplemental Table 1. Initial network toxicology evaluation from ProTox-3.0database**

| **Classification** | **Probability** |
| --- | --- |
| hERG Blockers | 0.007 |
| hERG Blockers (10um) | 0.023 |
| DILI | 0.277 |
| AMES Toxicity | 0.018 |
| Rat Oral Acute Toxicity | 0.013 |
| FDAMDD | 0.943 |
| Skin Sensitization | 0.744 |
| Carcinogenicity | 0.896 |
| Eye Corrosion | 0.994 |
| Eye Irritation | 0.997 |
| Respiratory | 0.995 |
| Human Hepatotoxicity | 0.734 |
| Drug-induced Nephrotoxicity | 0.999 |
| Drug-induced Neurotoxicity | 0.646 |
| Ototoxicity | 0.448 |
| Hematotoxicity | 0.089 |
| Genotoxicity | 0.033 |
| RPMI-8226 Immunitoxicity | 0.021 |
| A549 Cytotoxicity | 0 |
| Hek293 Cytotoxicity | 0.011 |
| BCF | 0.15 |
| IGC50 | 2.439 |
| LC50DM | 3.259 |
| LC50FM | 3.032 |
