# Supplementary material for: Environmental toxicant glyphosate induces cardiotoxicity: New insights from network toxicology, integrated machine learning, molecular modeling and multidimensional bioinformatics analysis
Source: Medicine (Baltimore). 2026 May 29;105(22):e48974. doi: 10.1097/MD.0000000000048974 (PMC13225543; doi:10.1097/MD.0000000000048974)
Supplement: Supplementary file 2 [file medi-105-e48974-s002.docx]

**Supplemental Table 2. Initial network toxicology evaluation from ADMETlab 3.0 database**

| **Classification** | **Target** | **Shorthand** | **Prediction** | **Probability** |
| --- | --- | --- | --- | --- |
| Organ toxicity | Hepatotoxicity | dili | Inactive | 0.93 |
| Organ toxicity | Neurotoxicity | neuro | Inactive | 0.84 |
| Organ toxicity | Nephrotoxicity | nephro | Active | 0.62 |
| Organ toxicity | Respiratory toxicity | respi | Active | 0.88 |
| Organ toxicity | Cardiotoxicity | cardio | Active | 0.50 |
| Toxicity end points | Carcinogenicity | carcino | Inactive | 0.58 |
| Toxicity end points | Immunotoxicity | immuno | Inactive | 0.99 |
| Toxicity end points | Mutagenicity | mutagen | Inactive | 0.90 |
| Toxicity end points | Cytotoxicity | cyto | Inactive | 0.68 |
| Toxicity end points | BBB-barrier | bbb | Active | 0.52 |
| Toxicity end points | Ecotoxicity | eco | Active | 0.61 |
| Toxicity end points | Clinical toxicity | clinical | Inactive | 0.57 |
| Toxicity end points | Nutritional toxicity | nutri | Inactive | 0.59 |
| Tox21-Nuclear receptor signalling pathways | Aryl hydrocarbon Receptor (AhR) | nr_ahr | Inactive | 0.97 |
| Tox21-Nuclear receptor signalling pathways | Androgen Receptor (AR) | nr_ar | Inactive | 0.99 |
| Tox21-Nuclear receptor signalling pathways | Androgen Receptor Ligand Binding Domain (AR-LBD) | nr_ar_lbd | Inactive | 0.99 |
| Tox21-Nuclear receptor signalling pathways | Aromatase | nr_aromatase | Inactive | 0.99 |
| Tox21-Nuclear receptor signalling pathways | Estrogen Receptor Alpha (ER) | nr_er | Inactive | 0.94 |
| Tox21-Nuclear receptor signalling pathways | Estrogen Receptor Ligand Binding Domain (ER-LBD) | nr_er_lbd | Inactive | 0.98 |
| Tox21-Nuclear receptor signalling pathways | Peroxisome Proliferator Activated Receptor Gamma (PPAR-Gamma) | nr_ppar_gamma | Inactive | 0.99 |
| Tox21-Stress response pathways | Nuclear factor (erythroid-derived 2)-like 2/antioxidant responsive element (nrf2/ARE) | sr_are | Inactive | 0.99 |
| Tox21-Stress response pathways | Heat shock factor response element (HSE) | sr_hse | Inactive | 0.99 |
| Tox21-Stress response pathways | Mitochondrial Membrane Potential (MMP) | sr_mmp | Inactive | 0.99 |
| Tox21-Stress response pathways | Phosphoprotein (Tumor Supressor) p53 | sr_p53 | Inactive | 0.99 |
| Tox21-Stress response pathways | ATPase family AAA domain-containing protein 5 (ATAD5) | sr_atad5 | Inactive | 0.99 |
| Molecular Initiating Events | Thyroid hormone receptor alpha (THRα) | mie_thr_alpha | Inactive | 0.75 |
| Molecular Initiating Events | Thyroid hormone receptor beta (THRβ) | mie_thr_beta | Inactive | 0.88 |
| Molecular Initiating Events | Transtyretrin (TTR) | mie_ttr | Inactive | 0.64 |
| Molecular Initiating Events | Ryanodine receptor (RYR) | mie_ryr | Inactive | 0.88 |
| Molecular Initiating Events | GABA receptor (GABAR) | mie_gabar | Inactive | 0.69 |
| Molecular Initiating Events | Glutamate N-methyl-D-aspartate receptor (NMDAR) | mie_nmdar | Active | 0.76 |
| Molecular Initiating Events | alpha-amino-3-hydroxy-5-methyl-4-isoxazolepropionate receptor (AMPAR) | mie_ampar | Inactive | 0.99 |
| Molecular Initiating Events | Kainate receptor (KAR) | mie_kar | Inactive | 0.99 |
| Molecular Initiating Events | Achetylcholinesterase (AChE) | mie_ache | Inactive | 0.81 |
| Molecular Initiating Events | Constitutive androstane receptor (CAR) | mie_car | Inactive | 1 |
| Molecular Initiating Events | Pregnane X receptor (PXR) | mie_pxr | Inactive | 0.66 |
| Molecular Initiating Events | NADH-quinone oxidoreductase (NADHOX) | mie_nadhox | Inactive | 0.70 |
| Molecular Initiating Events | Voltage gated sodium channel (VGSC) | mie_vgsc | Inactive | 0.81 |
| Molecular Initiating Events | Na+/I- symporter (NIS) | mie_nis | Inactive | 0.85 |
| Metabolism | Cytochrome CYP1A2 | CYP1A2 | Inactive | 0.99 |
| Metabolism | Cytochrome CYP2C19 | CYP2C19 | Inactive | 0.89 |
| Metabolism | Cytochrome CYP2C9 | CYP2C9 | Inactive | 0.86 |
| Metabolism | Cytochrome CYP2D6 | CYP2D6 | Inactive | 0.57 |
| Metabolism | Cytochrome CYP3A4 | CYP3A4 | Inactive | 0.99 |
| Metabolism | Cytochrome CYP2E1 | CYP2E1 | Inactive | 0.92 |
